# Evaluating Abdominal Obesity by Waist Circumference, Anthropometric Indices and Bioelectrical Impedance Analysis: A Comparative Pilot Study

**DOI:** 10.1002/osp4.70078

**Published:** 2025-06-20

**Authors:** Anastasiia Nahorna, Heiner Baur

**Affiliations:** ^1^ Department of Health Professions Discipline of Physiotherapy Bern University of Applied Sciences Bern Switzerland

**Keywords:** abdominal obesity (AO), bioelectrical impedance analysis (BIA), waist circumference (WC), waist‐to‐height ratio (WHtR), waist‐to‐hip ratio (WHR)

## Abstract

**Introduction:**

Abdominal obesity significantly increases the risk of various health conditions, making accurate assessment crucial for diagnosis and treatment. This study compares the effectiveness of anthropometric methods and conventional bioelectrical impedance analysis in evaluating abdominal obesity.

**Materials and Methods:**

Twenty adults (10 males, 10 females; age 45 ± 11.4 years; height 170 ± 8.63 cm; body weight 91.3 ± 19.2 kg; BMI 31.7 ± 5.31 kg/m^2^) participated in a single‐visit pilot study at the Bern Movement Lab at Bern University of Applied Sciences. Anthropometric measurements; including body weight, height, waist and hip circumferences; anthropometric indices; including BMI, waist‐to‐hip ratio, waist‐to‐height ratio and conventional bioelectrical impedance analysis were collected. Spearman's Rank Correlation was used for statistical analysis due to non‐normal data distribution.

**Results:**

Waist circumference, waist‐to‐hip ratio, and waist‐to‐height ratio consistently classified all participants as having abdominal obesity. In contrast, bioelectrical impedance analysis identified fewer cases, with only 40% of men and 10% of women classified as having abdominal obesity. Strong correlations were observed between waist circumference, waist‐to‐height ratio, and visceral fat, whereas waist‐to‐hip ratio showed weaker correlations.

**Conclusions:**

Simple anthropometric methods such as waist circumference, waist‐to‐hip ratio, and waist‐to‐height ratio are useful for evaluating abdominal obesity, with waist‐to‐height ratio often considered a more reliable predictor of central obesity. However, bioelectrical impedance analysis shows inconsistencies, and the waist‐to‐height ratio should be considered as a standard metric in future guidelines. Large‐scale multiethnic studies are recommended to validate these findings.

## 1 Introduction

Excessive abdominal fat mass (referred to as abdominal, central or visceral obesity) is strongly associated with a range of different disorders such as a variety of cancers [1, 2, 3], type‐2 diabetes [4, 5], fatty liver disease [6, 7], and cardiovascular mortality [8, 9]; it can affect bone health [10, 11] and provoke female infertility [12, 13, 14]. Even though there is no consensus regarding the protocol of waist (or abdominal) circumference (WC) measurement: World Health Organization (WHO) recommends the midpoint between the last palpable rib and the iliac crest [15], and the National Institutes of Health recommends the level of the umbilicus [16] and existing limitation for estimating the central obesity via WC among the different ethnical groups [17]—this parameter is recommended as the most practical anthropometric measurement for assessing patient's abdominal fat content by a number of the general recognized organizations [15, 16, 18, 19]. Although WC is meaningful on its own, some experts recognize other anthropometric parameters, which take body size or body shape into account ‐ waist‐to‐hip ratio (WHR) and waist‐to‐height ratio (WHtR)—as those which might be better clinical measures of central obesity [20, 21].

With increasing awareness of the risks produced by excessive abdominal fat mass, there is a growing demand for accurately validated tools for central obesity assessment applicable to clinical research and health care practice, as well as to homebased use for more effective prevention and treatment of abdominal obesity (AO). The bioelectrical impedance analysis (BIA) might be recognized as a possible one. The prevalence of BIA‐based devices for home use is growing but despite its simplicity, non‐invasiveness and affordable price, the current data about its accuracy lacks consistency [22, 23]. It can be hypothesized that the different considered reference methods lead to inconsistent evaluation of AO. Therefore, the aim was to compare the different mentioned methods for central obesity assessment.

## 2 Materials and Methods

### 2.1 Study Design and Subjects

Twenty individuals, 18–57 years of age (10 males; 10 females; age 45.05 ± 11.40 years; height 170.00 ± 8.63 cm; body weight 91.30 ± 19.20 kg; BMI 31.70 ± 5.31 kg/m^2^). Europids, were invited for a single visit to the Bern Movement Lab at Bern University of Applied Sciences. Inclusion criteria were: age between 18 and 60 years, WHR: ≥ 0.85 in females, ≥ 0.90 in males; WHtR: ≥ 0.50. Exclusion criteria were pathologies, diseases, injuries, surgeries, or pain limiting activities of daily living such as normal movements of the spine and lower extremities; known current pregnancy or breastfeeding mothers; psychological impairments to follow instructions; any comorbidities or circumstances limiting lifting capabilities; and inability to give consent. The study protocol was approved by the KEK Bern (Ethikkommission für die Forschung am Menschen, BASEC‐Nr: Req‐2022‐01421) and all participants gave written informed consent.

This study was designed as a pilot study to explore the feasibility of different anthropometric and BIA‐based approaches for assessing abdominal obesity. Due to its exploratory nature, no prior sample size calculation was conducted. Data were reviewed by the primary investigator. Demographic data such as age and sex were included. Participants underwent anthropometric evaluation and BIA on the same day.

### 2.2 Anthropometric Measurements

Anthropometric measurements included body weight, height, and waist and hip circumferences. Body weight was measured using a bioelectrical impedance analyzer (BIA, Tanita Europe GmbH, MC 780 MA P, Japan). Based on these, the following anthropometric indices were calculated: Body Mass Index (BMI), Waist‐to‐Hip Ratio (WHR), and Waist‐to‐Height Ratio (WHtR). Bioelectrical Impedance Analysis (BIA) was also conducted to assess visceral fat.

To minimize measurement errors, standardized measurement procedures were followed [15, 16, 24, 25]. Height was measured in centimeters (cm) from the bottom of the feet to the highest point of the head using a stadiometer (Seca 206, Seca, Hamburg, Germany). Waist and hip circumferences were measured in the standing position at the horizontal plane using a flexible, non‐stretchable plastic tape: for WC, at the midpoint between the lowest rib and the iliac crest (WHO, 15) and at the level of the umbilicus (NIH, 16), at the end of a normal expiration; for hip circumference, around the widest portion of the buttocks. Each measurement to the nearest 0.1 cm was taken twice, and the average was recorded. Ethnic‐specific cutoff values for WC (≥ 94 cm for men and ≥ 80 cm for women) were applied for central obesity evaluation [18].

WHR and WHtR were calculated manually based on WC measurements via WHO and NIH protocols properly. The WHR cutoff values of ≥ 0.95 for men and ≥ 0.80 for women were used as cutoff points for AO assessment according to the WHO protocol and the WHR cutoff values of ≥ 0.90 for men and ≥ 0.80 for women according to the NIH protocol (WHO 2008; NIH 1998). The cutoff values of AO assessment via WHtR were ≥ 0.50 [15]. BMI was calculated automatically by BIA (BIA, Tanita Europe GmbH, MC 780MA P, Japan). The recommended cutoff points for BMI (both by WHO and NIH) were taken for overweight and obesity status assessment within ≥ 25 kg/m^2^ and ≥ 30 kg/m^2^, respectively [15, 16].

### 2.3 Bioelectrical Impedance Analysis

The central obesity evaluation was estimated using the conventional BIA method (BIA, Tanita Europe GmbH, MC 780MA P, Japan) that has been extensively used in the number of epidemiological and clinical studies [26, 27, 28, 29, 30]. To achieve the normal conditions for BIA testing, participants were informed in advance about the following pretest requirements: no eating or drinking for 3 h before testing and no exercise for 12 h before the test. All participants confirmed adherence to these instructions. The measurement was performed between both hands and feet in a standing position with bare feet on the analyzer footplates. Each participant remained dressed in underwear, and the approximate weight of clothes was entered into the BIA Tanita (within 300 g for men and 400 g for women). Both hands were held stretched out a bit to give some room between the body and arms [31]. The standard (non‐athlete) mode, height, age, and sex were manually entered into the BIA Tanita manually. Using the Tanita's programming calculation, the parameters of body weight, fat percentage (%), BMI, and abdominal level score were recorded.

### 2.4 Statistical Analysis

The study's results were analyzed with Jamovi 2.3.28 software [32]. Mean, median, and standard deviation (SD) were calculated. The statistical analysis first checked the normal distribution of data through the Shapiro‐Wilk test. Considering the non‐normal distribution indicated by the Shapiro‐Wilk test, Spearman's Rank Correlation was applied to assess the relationship between WC, WHR, WHtR (both WHO and NIH protocols), and visceral fat estimated via BIA. A significance level of *p* < 0.05 was considered statistically significant for all analyses. Due to the exploratory nature of this pilot study and the limited sample size (*n* = 20), direct statistical comparisons between males and females, as well as between WHO and NIH methods, were not conducted. Instead, descriptive statistics and correlation analyses were applied to evaluate the relationships between obesity metrics and BIA parameters.

## 3 Results

### 3.1 Assessment of Normality in Anthropometric and Body Composition Measures

The normality of anthropometric and body composition measures was assessed using the Shapiro‐Wilk test, and the results are summarized in Table 1. The analysis was conducted separately for male and female participants to determine whether the data followed a normal distribution. Among the general anthropometric measures, body height showed no significant deviation from normality in both females (*W* = 0.92, *p* = 0.328) and males (*W* = 0.98, *p* = 0.949). However, body weight exhibited a non‐normal distribution in females (*W* = 0.84, *p* = 0.047), whereas in males, the deviation was not statistically significant (*W* = 0.88, *p* = 0.124). Similarly, BMI was normally distributed among females (*W* = 0.92, *p* = 0.321) but deviated from normality in males (*W* = 0.80, *p* = 0.015). In terms of body composition, visceral fat showed a significant departure from normality in both females (*W* = 0.82, *p* = 0.026), and males (*W* = 0.81, *p* = 0.021), indicating a skewed distribution. The hip circumference was normally distributed in females (*W* = 0.97, *p* = 0.914) but deviated significantly in males (*W* = 0.80, *p* = 0.013).

**TABLE 1:** The normality assumption through the Shapiro‐Wilk test.

	Gender	Body height, cm	Body weight, kg	BMI, units	Visceral fat, units	HC, cm	WC NIH, cm	WC WHO, cm	WHR WHO	WHR NIH	WHtR WHO	WHtR NIH
N	Female	10	10	10	10	10	10	10	10	10	10	10
	Male	10	10	10	10	10	10	10	10	10	10	10
Shapiro‐Wilk W	Female	0.92	0.84	0.92	0.82	0.98	0.90	0.96	0.94	0.83	0.86	0.96
	Male	0.98	0.88	0.80	0.81	0.80	0.83	0.86	0.97	0.96	0.90	0.86
Shapiro‐Wilk p	Female	0.328	0.047*	0.321	0.026*	0.914	0.250	0.845	0.505	0.031*	0.076	0.841
	Male	0.949	0.124	0.015*	0.021*	0.013*	0.031*	0.077	0.876	0.816	0.224	0.074

*Note:* Results present the Shapiro‐Wilk test statistics for assessing the normality of various anthropometric and body composition measures, including Body Height, Body Weight, BMI, Visceral Fat, Hip Circumference (HC), Waist Circumference (WC) as measured by WHO and NIH protocols, Waist‐to‐Hip Ratio (WHR) as measured by WHO and NIH protocols, and Waist‐to‐Height Ratio (WHtR) as measured by WHO and NIH protocols, separated by gender. The table includes the Shapiro‐Wilk W values and their corresponding *p*‐values for each measure. A *p*‐value below 0.05 indicates a significant deviation from normality. The sample size (N) indicates the number of participants in each gender group (*n* = 10 per group), with no missing data reported.

Abbreviations: BMI, body mass index; HC, hip circumference; WC, waist circumference; WHR, waist‐to‐hip ratio; WHtR, waist‐to‐height ratio.

*p < 0.05.

**p < 0.01.

***p < 0.001.

For waist circumference (WC) measurements based on WHO and NIH protocols, normality was largely upheld in females (*W* = 0.91, *p* = 0.250 for WC WHO; *W* = 0.97, *p* = 0.845 for WC NIH). However, in males, WC WHO exhibited a significant deviation from normality (*W* = 0.83, *p* = 0.031), whereas WC NIH was close to the normal threshold (*W* = 0.86, *p* = 0.077). The waist‐to‐hip ratio (WHR) based on the WHO protocol was normally distributed in both females (*W* = 0.94, *p* = 0.505) and males (*W* = 0.97, *p* = 0.876). However, the NIH‐based WHR showed significant deviation from normality in females (*W* = 0.83, *p* = 0.031), while it remained normally distributed in males (*W* = 0.96, *p* = 0.816). For waist‐to‐height ratio (WHtR), the WHO‐based measure showed borderline deviation in females (*W* = 0.86, *p* = 0.076) and was normally distributed in males (*W* = 0.90, *p* = 0.224). The NIH‐based WHtR remained normally distributed in both females (*W* = 0.97, *p* = 0.841) and males (*W* = 0.86, *p* = 0.074).

These findings suggest that some anthropometric and body composition variables, particularly visceral fat, BMI, hip circumference, and WC WHO in males, do not meet the assumption of normality. Given the non‐normal distribution of multiple variables, non‐parametric statistical tests were deemed more appropriate for subsequent analyses.

### 3.2 Descriptive Characteristics of the Study Participants

Table 2 presents the descriptive characteristics of the 20 study participants (10 males and 10 females). The dataset includes age, body height, body weight, waist circumference (WC) measured using WHO and NIH protocols, hip circumference (HC), BMI, and visceral fat levels. The mean and median values were calculated for each measurement. The mean age was 49.20 years (median: 53.00 years) for females and 40.90 years (median: 42.50 years) for males. The mean body height was 163.80 cm (median: 162.90 cm) for females and 175.80 cm (median: 175.30 cm) for males. The mean body weight was 81.30 kg (median: 86.20 kg) for females and 101.30 kg (median: 97.80 kg) for males. For waist circumference, the WHO protocol reported a mean of 99.00 cm (median: 99.50 cm) for females and 107.60 cm (median: 103.80 cm) for males, while the NIH protocol showed slightly higher values with a mean of 103.30 cm (median: 105.30 cm) for females and 111.00 cm (median: 107.50 cm) for males. The mean hip circumference was 107.00 cm (median: 107.00 cm) for females and 110.00 cm (median: 107.00 cm) for males. The BMI values indicated that females had a mean of 30.40 kg/m^2^ (median: 30.10 kg/m^2^), while males had a slightly higher mean of 33.00 kg/m^2^ (median: 31.00 kg/m^2^). Additionally, visceral fat levels were 8.60 units (median: 8.00 units) for females and 13.10 units (median: 10.50 units) for males.

**TABLE 2:** Descriptive characteristics of the study participants’ results present the descriptive statistics for general characteristics, anthropometric measurements, and anthropometric indices categorized by gender.

Category	Variable	Female (*n* = 10)	Male (*n* = 10)
General characteristics	Age (years)	49.20 ± 9.15 (median: 53.00)	40.90 ± 12.37 (median: 42.50)
Anthropometric measurements	Body height (cm)	163.80 ± 4.76 (median: 162.90)	175.80 ± 7.43 (median: 175.30)
	Body weight (kg)	81.30 ± 9.34 (median: 86.20)	101.30 ± 21.66 (median: 97.80)
	Waist circumference WHO (cm)	99.00 ± 7.07 (median: 99.50	107.60 ± 12.64 (median: 103.80)
	Waist circumference NIH (cm)	103.30 ± 8.18 (median: 105.30)	111.00 ± 15.42 (median: 107.50)
	Hip circumference (cm)	107.00 ± 5.72 (median: 107.00)	110.00 ± 14.30 (median: 107.00)
Anthropometric indices	BMI (kg/m^2^)	30.40 ± 3.66 (median: 30.10)	33.00 ± 6.51 (median: 31.00)
BIA method	Visceral fat, units	8.60 ± 1.78 (median: 8.00)	13.10 ± 7.72 (median: 10.50)

*Note:* The measures include age (years), body height (cm), body weight (kg), hip circumference (cm), waist circumference (cm) as measured by WHO and NIH protocols, BMI (kg/m^2^), and visceral fat (units). The mean, median, and standard deviation were calculated for each group. The sample size (N) indicates the number of participants in each category.

### 3.3 Evaluation of Abdominal Obesity Using WHO, NIH, and BIA Methods

Table 3 presents the descriptive statistics for abdominal obesity (AO) evaluation categorized by gender and measurement method, including Waist Circumference (WC, cm) measured using WHO and NIH protocols, Waist‐to‐Hip Ratio (WHR), Waist‐to‐Height Ratio (WHtR), and Visceral Fat (units) assessed by BIA. Among females with AO status (*n* = 10), the mean WC was 99.0 cm (WHO) and 103.0 cm (NIH), WHR was 0.91 (WHO) and 0.96 (NIH), and WHtR was 0.59 (WHO) and 0.63 (NIH). Similarly, among males with AO status (*n* = 10), the mean WC was 108.0 cm (WHO) and 111.0 cm (NIH), WHR was 0.98 (WHO) and 1.01 (NIH), and WHtR was 0.61 (WHO) and 0.63 (NIH). According to BIA visceral fat assessment, 1 female living with AO had a mean visceral fat level of 12.0 units, while 4 males living with AO had a mean visceral fat level of 19.8 units. In the group without AO, 9 females and 6 males had mean visceral fat levels of 8.2 and 8.6 units, respectively. These findings highlight differences in abdominal obesity assessment based on WHO and NIH criteria and demonstrate the variation in visceral fat levels between individuals living with and without AO.

**TABLE 3:** Summary of abdominal obesity evaluation using WHO and NIH measurements protocols and BIA methods.

Category	Variable	AO status	WHO female (mean ± SD, N)	WHO male (mean ± SD, N)	NIH female (mean ± SD, N)	NIH male (mean ± SD, N)
Anthropometric measurements	WC, cm	AO	99.0 ± 7.07, *n* = 10	108.0 ± 12.60, *n* = 10	103.00 ± 8.18, *n* = 10	111.00 ± 15.40, *n* = 10
		None				
Anthropometric indices	WHR	AO	0.91 *n* = 10	0.98 *n* = 10	0.96 *n* = 10	1.01 *n* = 10
		None				
	WHtR	AO	0.59 *n* = 10	0.61 ± 0.1, *n* = 10	0.63 *n* = 10	0.63 ± 0.8, *n* = 10
		None				
BIA method	Visceral fat, units		Female (mean ± SD, N)	Male (mean ± SD, N)		
		AO	12.0, *n* = 1	19.80 ± 8.60, *n* = 4		
		None	8.20 ± 1.40, *n* = 9	8.6 ± 2,00 *n* = 6		

*Note:* Results present the descriptive statistics for anthropometric measurements, anthropometric indices, and visceral fat analysis, categorized by gender and abdominal obesity (AO) status. The measures include Waist Circumference (WC, cm) as measured by WHO and NIH protocols, Waist‐to‐Hip Ratio (WHR), Waist‐to‐Height Ratio (WHtR), and Visceral Fat (units) measured by BIA. The mean, standard deviation (SD), and sample size (N) were calculated for each category.

### 3.4 The Spearman's Rank Correlation Analysis

Table 4 presents the Spearman's Rank Correlation analysis assessing the relationships between Waist Circumference (WC) as measured by WHO and NIH protocols, Waist‐to‐Hip Ratio (WHR) based on WHO and NIH criteria, Waist‐to‐Height Ratio (WHtR) from WHO and NIH protocols, and Visceral Fat levels. A positive correlation was observed between WC WHO and WC NIH (*ρ* = 0.87, *p* < 0.05) and between WHtR WHO and WHtR NIH (*ρ* = 0.86, *p* < 0.05). WHR WHO and WHR NIH were moderately correlated (*ρ* = 0.74, *p* < 0.05). WHR NIH correlated with visceral fat (*ρ* = 0.51, *p* < 0.05), while WHR WHO had a lower correlation with visceral fat (*ρ* = 0.32, *p* > 0.05). WC WHO and WC NIH correlated with visceral fat (*ρ* = 0.82 and *ρ* = 0.81, respectively, both *p* < 0.05). WHtR WHO showed a correlation with visceral fat (*ρ* = 0.59, *p* > 0.05).

**TABLE 4:** Correlation between the different methods of the central obesity evaluation (Spearman's Rank Correlation).

	WC WHO	WC NIH	WHR WHO	WHR NIH	WHtR WHO	WHtR NIH	Visceral fat
WC WHO	—	0.87*	0.52*	0.64**	0.86*	0.77*	0.82*
WC NIH	0.87*	—	0.26	0.60**	0.75*	0.79*	0.81*
WHR WHO	0.52*	0.26	—	0.74*	0.49*	0.11	0.32
WHR NIH	0.64**	0.60**	0.74*	—	0.52*	0.42	0.51*
WHtR WHO	0.86*	0.75*	0.49*	0.52*	—	0.86*	0.59
WHtR NIH	0.77*	0.79*	0.11	0.42	0.86*	—	0.58
Visceral fat	0.82*	0.81*	0.32	0.51*	0.59	0.58	—

*Note:* Results present the Spearman's rank correlation coefficients (rho) for various anthropometric measures, including Waist Circumference (WC) as measured by WHO and NIH protocols, Waist‐to‐Hip Ratio (WHR) as measured by WHO and NIH protocols, Waist‐to‐Height Ratio (WHtR) as measured by WHO and NIH protocols, and Visceral Fat levels. The table displays the correlation coefficients (Spearman's rho) in the upper triangle and *p*‐values in the lower triangle, indicating the strength and significance of the relationships between these measures. Higher rho values suggest stronger correlations, while *p*‐values determine statistical significance.

Abbreviations: WC, waist circumference; WHR, waist‐to‐hip ratio; WHtR, waist‐to‐height ratio.

*p < 0.05.

**p < 0.01.

***p < 0.001.

## 4 Discussion

This study aimed to compare the various anthropometric measures and conventional BIA for central obesity evaluation in a male and female cohort. In this pilot study, direct statistical comparisons between males and females were not conducted due to the limited sample size (*n* = 20), which restricts the statistical power necessary for detecting significant differences. Instead, descriptive statistics and correlation analyses were used to evaluate the relationships between obesity metrics and BIA parameters. Future studies with larger and more diverse populations are needed to confirm potential gender differences in abdominal obesity assessment.

The findings revealed that the use of WHO and NIH WC measurement protocols, along with associated indexes such as WHR and WHtR, consistently classified all 10 male and all 10 female participants as having AO, highlighting a high prevalence of AO within these groups. However, the BIA method identified only 40% of the male participants and 10% of the female participants classified as having AO. This discrepancy suggests that visceral fat measurements via BIA do not align as closely with widely accepted methods for assessing AO like WC, WHR, and WHtR.

Although BIA is used to estimate AO in epidemiological and clinical studies [26, 27, 28, 29, 30], our results showed inconsistent correlations with key anthropometric measures, particularly WHR, and its indirect nature of measurement may fail to accurately capture the localized distribution of fat, especially around the central body or within the trunk.

Given these findings, BIA appears less reliable as a stand‐alone method for assessing AO. While it may provide supplementary information, its limitations suggest that reliance on BIA alone could lead to underestimations or misclassifications of abdominal obesity. The findings partially align with the research by LD Kawaji & JA Fontanilla [30], particularly in recognizing WC measurement, both per WHO and NIH protocols, as a highly sensitive tool for evaluating AO. However, they also diverge from their conclusion that suggests BIA as a more relevant method for assessing AO [30]. This discrepancy highlights the need to be cautious when applying these results broadly, especially given the ethnic diversity of populations.

Notably, the data revealed slightly higher mean values for WC obtained via the NIH protocol compared to the WHO protocol (e.g., for males, 111 vs. 108 cm; for females, 103.3 vs. 99 cm). These findings contradict the conclusions of other authors and authorities, such as Croft et al. [33] and WHO [9], who suggested that WC measurements taken at the level of the umbilicus (as via NIH protocol) may underestimate the true WC compared to measurements taken at the midway point between the lowest rib and the iliac crest (as via WHO protocol). Despite using different tape placements for WC measurements, both WHO and NIH protocols utilize the same ethnic‐specific cutoff values for assessing AO (≥ 94 cm for men and ≥ 80 cm for women, as per the International Diabetes Federation) [17]. Our data analysis revealed that, despite the differing measurement techniques, all participants were consistently classified as having AO using both the WHO and NIH protocols.

While WHtR has been studied and proposed by various researchers as a potentially accurate indicator of central obesity and associated health risks [20, 21, 34], it has not been officially adopted by WHO or NIH as a standard metric for assessing AO in their global or national guidelines. However, with the analysis provided in this study, it might be assumed that WHtR is a consistent and reliable metric for the assessment of AO across both male and female participants. The data show that WHtR, when measured using both WHO and NIH protocols, uniformly classified all participants as having AO, closely aligning with the results from WC and WHR. The consistency of WHtR in identifying AO, supported by its strong correlations with both WC and WHR, underscores its accuracy as an indicator of AO. Although the standard deviations for WHtR—0.137 for the WHO protocol and 0.0860 for the NIH protocol in males, and 0.0374 for the WHO protocol and 0.0512 for the NIH protocol in females—indicate some variability, WHtR should be considered as an effective and reasonably consistent measure for assessing AO in diverse populations.

Taken together, the findings of this study suggest that simple and widely accessible anthropometric methods such as WC and WHR, in accordance with WHO and NIH guidelines, are effective in AO evaluation. Conversely, BIA measurements demonstrate inconsistencies when compared to these established methods, indicating that BIA should not be relied upon for the accurate assessment of central obesity. Given its demonstrated consistency and reliability in this study, WHtR should be considered for inclusion as a standard metric for assessing abdominal obesity in future guidelines.

## Author Contributions

Anastasiia Nahorna and Heiner Baur have made the same contributions to the conception and the design of the study and to the acquisition, analysis, and interpretation of the data. Both authors have participated in drafting the manuscript. Both authors have read and approved the final version of the manuscript.

## Conflicts of Interest

The authors declare no conflicts of interest.
